# English Football Players are not as Bad at Kicking Penalties as Commonly Assumed

**DOI:** 10.1038/s41598-020-63889-6

**Published:** 2020-04-27

**Authors:** Michel Brinkschulte, Philip Furley, Daniel Memmert

**Affiliations:** German Sport University Cologne, Institute of Training and Computer Science in Sport, Am Sportpark Müngersdorf 6, Köln, 50933 Germany

**Keywords:** Psychology, Human behaviour

## Abstract

The previous performance of the English men’s national football team in penalty shootouts has led to the widespread stereotype that English football players are particularly bad at scoring penalties. Research has proposed possible reasons behind this alleged “penalty curse”. When looking at these reasons, the question arises if English football players per se have trouble scoring penalty kicks. Therefore, we analyzed the performance of a large sample of penalty takers during all World- and European Championships (*N* = 696) and, additionally, in some of the highest European leagues over a ten-year period (*N* = 4,708). The results reveal no significant differences between the success rates (on average between 71–79%, depending on the type of penalty kick and on the type of competition) of penalty takers from different nations. Therefore, we conclude that English players perform as well as players from other nations and that poor performance in penalties lay beyond the factor nationality.

## Introduction

Penalty kicks play an important role in association football (soccer) and will likely play an important role in upcoming events like the UEFA European Championship 2021. UEFA introduced penalty shootouts to major tournaments in 1976 (FIFA followed in 1978) as a means of deciding matches in the knockout phase of major tournaments when the score is a draw at the end of the match. These shootouts have decided 18 UEFA European Championship matches including one for winning the tournament (1976) as well as 30 FIFA World Cup matches including two for winning the World Cup trophy (1994 and 2006) and can be considered as the pinnacle of high-pressure performance in football^1,2^. Pertinent to the present research, studies have suggested that not all nations perform equally well in these high-stakes penalty situations^3–5^. One specific result from this research has received an extraordinary amount of public attention, which has led to a well-known stereotype: English football players are extremely bad at shooting penalties. It is, in fact, nearly impossible to find opposing opinions about this stereotype in the media, and even acknowledged football experts in television commentaries openly talk about how bad England are at penalties – an opinion that vast amounts of newspaper and magazine reports seemingly support^6–9^.

As is often the case with stereotypes, there may be some truth behind it. A brief look at the numbers of the penalty shootouts involving the England national team does in fact show a negative balance. Since 1978, the England men’s national team has only won three out of the nine shootouts they participated in (losses: World Cups in 1990, 1998 and 2006; European Championships in 1996, 2004, and 2012). This negative record of accomplishment has led scientists to seek for potential explanations. About a decade ago, Norwegian sport psychologist Geir Jordet investigated the reasons behind the alleged “English penalty curse”. In general, Jordet and colleagues demonstrated that multiple variables seem to influence the general success of a penalty kick taken by any player, including psychological factors, physiological factors, skill, and chance (for in depth information see^5^). Relevant to the present research, Jordet argued that players who enjoy high international esteem (defined as receiving one or more prestigious international awards), perform worse than players with lower levels of public status in penalty shootouts. The fact that several of the England national team players (in the sample investigated) have received these awards could potentially explain a negative performance in penalty shootouts to some extent^3,4^. This finding suggests that it is not the nationality of a player per se that influences the outcome of a penalty shot, but rather confounding variables (like an individual player’s status) with a player’s nationality that affect success in penalty shootouts. A further problem with the studies that purportedly backed up the stereotype that English players are bad at penalties is that sample sizes in these studies were small. Given the importance of sample sizes in reproducible research^10–12^, in our opinion it remains questionable if English players are in fact worse penalty takers compared to players from other nations. Therefore, we sought to return to this question by investigating penalty success as a function of player nationality. First, we analyzed all penalty kicks taken during World- and European Championships (696 penalties). Further, we analyzed a large sample of penalty kicks taken in major European leagues (4,708 penalties).

We consider this research important, as a different line of study has highlighted the influence of stereotypes on sporting performance, even if the stereotypes are not true. This research suggests that merely introducing a negative stereotype about a social group can potentially result in performance decrements of members of that group; a finding that has been labeled *stereotype threat*^13^. Various studies in the sports domain have found evidence for detrimental effects of stereotypes in sports^14,15^. A recent study showed that stereotypes could also have debilitating effects on learning motor skills^16^, for example due to not trying as hard^17^. Therefore, we consider it theoretically feasible that the persistence of the negative stereotype of “English football players being bad at penalties” might act as a kind of self-fulfilling prophecy and contribute to English players actually performing worse at penalties.

Taken together, we consider it important to investigate if English football players per se are actually as bad at penalties as stated in the media. If this was the case, then the under-average performance of the England national team players in penalty shootouts should be evident when contrasting the performance of English penalty takers with penalty takers from other nations. A contrary outcome would speak against the widespread stereotype that English players are bad at penalty kicks. To test this, we investigated the performance of penalty takers and their rate of success in all penalties taken during European and World Cup matches as well as all penalty kicks taken over a ten-year period in some of the highest European leagues.

## Method

First, we sampled all penalty kicks taken in penalty shootouts and during the game in European and World Cup matches since 1976. Second, we sampled all penalty kicks taken in the highest German, English, Spanish, Italian, and Dutch leagues from the seasons 2006/2007 until inclusively 2015/2016. We collected the data from various websites that provide information on the different competitions, player profiles, penalty shootouts, and in-game penalty kicks (e.g., soccerstats.com, wikipedia.org, thestatszone.com, fifa.com, uefa.com, transfermarkt.de). Along with listing the players’ names and the dates of the seasons, we coded each penalty kick as either successful or unsuccessful. The coded penalties were double-checked using multiple sources. We then used this information to calculate a success rate for each individual player. Additionally, we coded the nationality of all penalty takers. The independent variable was the nationality of the penalty taker. The dependent variable was the percentage of scored penalties of the respective player. In the first analyses of World- and European Championships, we distinguished between performance in penalty shootouts and performance in in-game penalties.

Data were analyzed with univariate ANOVAs and Bonferroni corrected pairwise comparisons. The level of significance was set at 0.05. Partial eta-squared effect sizes were calculated and interpreted in line with statistical convention as 0.01 being a small effect, 0.06 a medium effect, and 0.14 a large effect. In addition, we calculated one-sample t-tests comparing the performance of every nation to the mean of the entire sample. If assumptions of the parametric tests were violated, we conducted non-parametric tests (i.e., Mann-Whitney-U-Tests).

## Results

### World- and European Championships

#### Penalty shootouts

Within penalty shootouts, 387 different players kicked 473 penalties. On average, the players scored 71.97 percent of the penalties (*SD* = 43.2). A univariate ANOVA did not reveal a main effect for the factor nationality on the percentages of scored penalties (*F*[6, 380] = 0.985, *p* = 0.435, *η*² = 0.015). Although the effect was non-significant, it can be considered a small effect by statistical convention. None of the Bonferroni corrected pairwise comparisons revealed significant differences between any of the analyzed nations (all *p* > 0.64). Hence, there were no significant differences between the success rates of penalty takers from different nations. Only Germany significantly (85.29%; *SD* = 35.94; (*t*[33] = 2.161*, p* = 0.038, two-tailed) differed from the overall sample mean of 71.97 percent. None of the other nations significantly differed from the overall sample mean: England (61.32%; *SD* = 45.83; (*t*[27] = *−*1.230*, p* = 0.229, two-tailed); Spain (67.83%; *SD* = 46.70; (*t*[28] = *−*0.478*, p* = 0.637, two-tailed); Italy (69.00%; *SD* = 43.58; (*t*[42] = −0.447*, p* = 0.657, two-tailed); Netherlands (67.39%; *SD* = 44.23; (*t*[22] = −0.496*, p* = 0.625, two-tailed); Brazil (68.75%; *SD* = 47.87; (*t*[15] = *−*0.269*, p* = 0.792, two-tailed); Others (73.14%; *SD* = 42.86; (*t*[213] = 0.398*, p* = 0.691, two-tailed).

#### In-game penalties

Within in-game penalties, 159 different players kicked 223 penalties. On average, the players scored 78.74 percent of the penalties (*SD* = 37.6). A univariate ANOVA did not reveal a main effect for the factor nationality on the percentages of scored penalties (*F*[6, 152] = 0.394, *p* = 0.882, *η*² = 0.015). Although the effect was non-significant, it can be considered a small effect by statistical convention. None of the Bonferroni corrected pairwise comparisons revealed significant differences between any of the analyzed nations (all *p* > 0.98). Hence, there were no differences between the success rates of penalty takers from different nations. None of the countries differed from the overall sample mean of 78.74 percent: England (90.00%; *SD* = 22.36; (*t*[4] = 1.126*, p* = 0.323, two-tailed); Germany (75.00%; *SD* = 41.83; (*t*[5] = −0.219*, p* = 0.835, two-tailed); Spain (77.43%; *SD* = 31.07; (*t*[13] *= −*0.158*, p* = 0.877, two-tailed); Italy (65.00%; *SD* = 47.43; (*t*[9] *=* −0.916*, p* = 0.384, two-tailed); Netherlands (75.00%; *SD* = 46.29; (*t*[7] *=* −0.229*, p* = 0.826, two-tailed); Brazil (87.5%; *SD* = 35.35; (*t*[7] *=* 0.701*, p* = 0.506, two-tailed); Others (79.49%; *SD* = 37.75; (*t*[107] = 0.207*, p* = 0.837, two-tailed).

#### Comparison of shootout and in-game penalties

**Figure 1:**
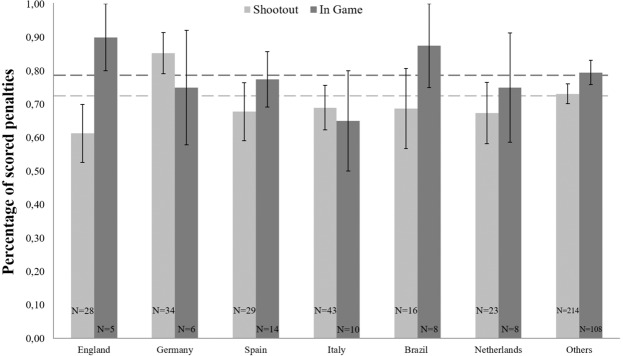
Mean percentages of scored penalty kicks in World- and European Championships as a function of nationality and type of penalty kick. The *N*’s refer to the number of players analyzed for each nation (387 players kicked 473 penalties in shootouts and 157 different players kicked 223 in-game penalties in the analyzed period). Error bars represent standard errors of the mean.

Figure 1 shows the percentages of scored penalties as a function of nationality of the penalty takers and the type of penalty (shootout vs. in-game). Although Fig. 1 shows some interesting descriptive trends (e.g., English players performing on average almost 30 percent worse in shootouts than in in-game penalties; Germany performing 10 percent better in shootouts than in-game penalties), a 2 (type-of-penalty) × 7 (nationality) ANOVA neither revealed a main effect for the factor type-of-penalty (*F*[1, 532] = 1.786, *p* = 0.182, *η*² = 0.003) nor an interaction between type-of-penalty and nationality (*F*[6, 532] = 0.508, *p* = 0.803, *η*² = 0.006). Therefore, overall penalty performance did not significantly differ for in-game penalties and penalties during shootouts. Neither did any of the analyzed nations significantly perform better or worse depending on the type of penalty. The latter result was confirmed by a series of nonparametric Mann-Whitney-U-Tests for all nations (England, *p* = 0.268; Germany, *p* = 0.541; Spain, *p* = 0.916; Italy, *p* = 0.822; Netherlands, *p* = 0.674; Brazil, *p* = 0.490; and others, *p* = 0.315).

### European Leagues

**Figure 2:**
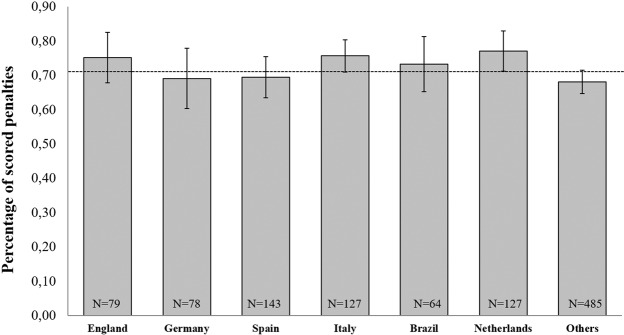
Mean percentages of scored penalty kicks in some of the highest European leagues as a function of nationality. The *N*’s refer to the number of players analyzed for each nation (altogether 1,103 players kicked 4,708 penalties in the analyzed period). Error bars represent standard errors of the mean.

Within this sample, 1,103 different players shot 4,708 penalties of which 71.01% were scored (*SD* = 35.4). Figure 2 shows the percentages of scored penalties as a function of nationality of the penalty takers.

A univariate ANOVA did not reveal a main effect for the factor nationality on the percentages of scored penalties (*F*[6, 1096] = 1.875, *p* = 0.082, *η*² = 0.010). Although the effect was non-significant, it can be considered a small effect by statistical convention. None of the Bonferroni corrected pairwise comparisons revealed significant differences between any of the analyzed nations (all *p* > 0.21). Hence, there were no differences between the success rates of penalty takers from different nations. Only the Netherlands (and Italy tentatively *p* = 0.053) significantly differed from the overall sample mean of 71.01%. However, this significant difference does not hold when correcting for multiple tests. None of the other nations significantly differed from the overall sample mean: England (75.14%; *SD* = 32.98; (*t*[78] = 1.115*, p* = 0.268, two-tailed); Germany (69.03%; *SD* = 39.03; (*t*[77] = *−*0.445*, p* = 0.658, two-tailed); Spain (69.41%; *SD* = 35.98; (*t*[143] = *−*0.530*, p* = 0.597, two-tailed); Italy (75.61%; *SD* = 26.56; (*t*[126] *=* 1,957*, p* = 0.053, two-tailed); Netherlands (77.10%; *SD* = 33.38; (*t*[126] *= 2*.061*, p* = 0.041, two-tailed); Brazil (73.20%; *SD* = 32.23; (*t*[64] *=* 0.545*, p* = 0.588, two-tailed); Others (68.03%; *SD* = 37.66; (*t*[484] = −1.735*, p* = 0.083, two-tailed).

## General Discussion

The aim of the present research was to determine if the nationality of a player influences his success in taking penalty kicks and, thereby, shed light on the prevalent stereotype that English football players perform poorly in shooting penalty kicks. The simple answer to this research question is that the empirical data do not support this stereotype. Although the effect of player nationality never reached statistical significance in the overall ANOVA, the partial eta-squared effect sizes ranged between 0.01–0.015 and can be considered a small effect by statistical convention of player nationality on penalty success rate. However, concerning our main research question, English players scored as many penalties as players with a comparable skill level from different nations. Hence, it would be inaccurate to tie the prevalence of lost penalty shootouts by the English men’s national team to the nationality of the players, as largely assumed by the media (e.g.^6–9^). Our results implicate that there are no significant differences between the success rates of penalty takers from different nations.

At a descriptive level of analyses, English players performed worse in shootouts (61.32%) than in in-game penalties during World- and European Championships (90.00%) and European leagues (75.14%). Compared to the respective sample means, English players slightly over-performed in in-game penalties and underperformed in shootouts. However, none of these comparisons reached statistical significance. Based on these findings, we conclude that the factor nationality does not explain meaningful variance in penalty performance and the explanation for the poor performance in penalty shootouts of the England national team in the past most likely lies with other factors including the unreliable measurement of penalty performance^18^. Moreover, it is likely that cognitive biases like the availability heuristic^19^ lead people to overgeneralize based on salient (e.g., emotional) events that readily come to mind (e.g., a loss in an important penalty shootout). This cognitive bias might contribute to the stereotype that English players are bad at penalty kicks as a loss in an emotional penalty shootout during a World or European Cup arguably comes more easily to mind than an in-game penalty. In turn, people believe that English players miss more penalties than they actually do.

Nevertheless, we do not want to exclude the possibility that there might be some meaningful differences between nations when it comes to penalties. For example, media reports have suggested that English players might be more afraid of the brutal English boulevard press when it comes to penalty shootouts, which might lead them to be more inclined to “choke under pressure”^20^. It has also been suggested that the more prevalent belief amongst English coaches and players that penalty shootouts are a lottery and their outcome is simply due to chance has caused the national team to not prepare adequately for these events, potentially contributing to poorer performance^21^. In this respect, other nations like Germany that have performed above average in high-pressure shootouts might prepare more effectively for these unique events. This poses the question of how teams and players can optimally prepare for these events. While this question is beyond the scope of the present research, several recommendations in this respect have been made in the literature^1,2,21^ and more future research is needed to determine the effectiveness of applied interventions on scoring penalties and winning shootouts (such as^22,23^).

In the future, there will certainly be further penalty shootouts determining the success of the England national team in major competitions. Perhaps, with the help of scientific findings, practice, or merely an increasing number of penalty shootouts, their record of accomplishment will even out – and the widespread stereotype that English football players are bad at scoring penalty kicks will eventually fade.

## Data Availability

The datasets generated and analyzed during the current study are available from the corresponding author on reasonable request.
